# A WeChat-Based Self-Management Intervention for Community Middle-Aged and Elderly Adults with Hypertension in Guangzhou, China: A Cluster-Randomized Controlled Trial

**DOI:** 10.3390/ijerph16214058

**Published:** 2019-10-23

**Authors:** Xiaowen Li, Tong Li, Jianying Chen, Yuanling Xie, Xia An, Yunhong Lv, Aihua Lin

**Affiliations:** 1 Department of Medical Statistics, School of Public Health, Sun Yat-sen University, Guangzhou 510080, China; 2 Baiyun Community Healthcare Center, Guangzhou 510080, China; 3 Department of Health Service and Management, Xinhua College of Sun Yat-sen University, Guangzhou 510080, China

**Keywords:** hypertension, mHealth, intervention, self-management

## Abstract

This study aimed to assess whether a WeChat-based self-management intervention would be effective for community middle-aged and elderly adults with hypertension in Guangzhou, China. We conducted a cluster-randomized control trial with a total of 464 participants (intervention, *n* = 186; control, *n* = 276) between March 2018 and May 2019. The self-management intervention lasted for 6 months, consisting of health education, health promotion, group chat, and blood pressure (BP) monitoring. All individuals in the baseline and follow-up surveys were assessed for BP and completed a hypertension knowledge questionnaire, self-efficacy scale, self-management scale, and social support scale. A total of 253 participants (intervention, *n* = 110; control, *n* = 143) completed the follow-up survey and were included in the analysis. The adjusted mean differences in the changes in systolic blood pressure (SBP) and diastolic blood pressure (DBP) between the intervention and control groups were −6.9 (95% Confidence Interval (CI) −11.2 to −2.6; *p* = 0.002) and −3.1 (95% CI −5.7 to −0.6; *p* = 0.016) mmHg, respectively. Individuals who participated in the intervention program had better BP monitoring, improved their hypertension self-management as well as parts of their disease knowledge and self-efficacy. The WeChat-based self-management intervention may be a feasible and efficient program to help Chinese community middle-aged and elderly hypertensive patients lower BP and improve self-management.

## 1. Introduction

Hypertension has become one of the most important causes of disease burden worldwide, as it is considered the leading risk factor for cardiovascular diseases [1]. In China, high systolic blood pressure (SBP) was the top risk factor for the number of deaths in 2017 [2], accounting for 2.54 million deaths. The latest China Hypertension Survey [3] showed that the prevalence rate of hypertension was rising rapidly, and 23.2% of adults (at least 18 years of age) had hypertension. Despite the rate of hypertension control rising from 6.1% (2002) to 16.8% (2015) [4], it is still at a low level.

Improving self-management (e.g., increasing medication adherence, enhancing monitoring blood pressure (BP), and reducing salt intake) is an effective way [5] to reduce BP, but most patients’ knowledge of self-management and confidence in their ability to self-manage are not sufficient. Studies [6,7,8] have demonstrated that health education or other health promotion interventions could help improve self-management and lower BP. However, given the large number of hypertensive patients in China, a traditional in-person intervention would be very costly. A more convenient, low-cost, and effective intervention is needed. In recent years, with the popularity of mobile phones, mobile health (mHealth) interventions have become more popular as new solutions for various diseases. For example, the intervention of home BP telemonitoring and pharmacist management achieved a 10.7 (95% CI 7.3–14.3) mmHg decrease in SBP compared with usual care [9]. However, only a few of the existing mHealth studies have provided whole self-management interventions (most solely focus on home BP monitoring). In addition, because different interventions use different platforms and contents, the outcomes are varied [9,10,11].

There are more than 100 apps supporting self-management (e.g., logging BP, lifestyle advice, and hypertension information) for hypertensive patients in app stores around the world [12,13]. However, these apps have no evidence to confirm their effectiveness and lack a clear theoretical basis to ensure their security. In addition, because they are completely new apps with a single function, they are not easy for middle-aged and older Chinese adults to use, and they lack flexible responses (e.g., personalized advice and real-time health counseling services). WeChat is the most popular social network (like Facebook and Twitter) in China, having more than 1.13 billion monthly active users in the 2nd quarter of 2019 [14]. It offers voice and text messaging, group chats, subscriptions, applets, and more features, all in a single app. Due to its large user groups and multiple functions, WeChat has been widely used as a more flexible technological tool for disease management [15,16]. It can also deliver to hypertensive patients as a more cost-effective intervention for community chronic disease management. Some studies [17,18] have mentioned the use of WeChat in hypertension self-management, but its function has not yet been assessed.

There are few such intervention studies in China, most of which deliver short messages only. Therefore, our study assessed whether a WeChat-based self-management intervention could be a feasible and effective way to help community middle-aged and elderly adults with hypertension reduce BP in Guangzhou, China.

## 2. Materials and Methods

### 2.1. Study Design and Participants

The WeChat-based self-management intervention study was a prospective, cluster-randomized controlled trial between March 2018 and May 2019. Potential participants registered in the community who came for clinic visits were identified by trained health workers in two community health care centers in Yuexiu District, located in the center of Guangzhou, China.

Individuals who were WeChat users, who were aged 45–70 years, who had lived in the community for at least six months, who reported a definite diagnosis of hypertension while taking or having ever taken antihypertensive drugs, and who were willing to participate in our study were eligible for recruitment. Exclusion criteria were a severe cognitive disorder, serious clinical complications, a known cause of secondary hypertension, or already participating in other similar programs within a year. All participants signed informed consent forms. This study protocol was approved by the Institutional Review Board of the School of Public Health, Sun Yat-sen University.

### 2.2. Randomization

To avoid contamination by participants from the same community, we took one community health service center as a cluster in the randomization. There are 18 community health service centers with residents having similar characteristics in Yuexiu District. We randomly chose two centers and divided Baiyun Street as an intervention group and Dadong Street as a control group via SPSS version 25.0 (IBM Corp, Armonk, NY, United States).

### 2.3. Intervention Program

**Figure 1:**
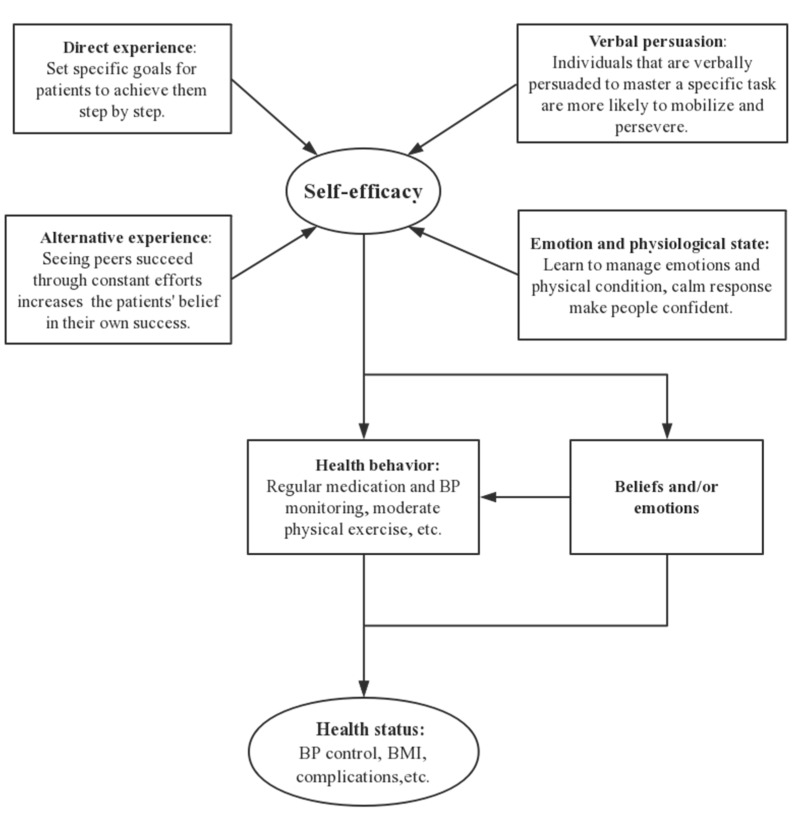
The framework of self-efficacy in this study. BP: blood pressure.

The six-month intervention program was guided by the self-efficacy theory [19,20] to improve the self-management of participants. The self-efficacy theory notes that direct experience, alternative experience, verbal persuasion, and emotion and physiological state could help to improve self-efficacy and promote healthy behaviors and beliefs/emotions to achieve an improved health state (Figure 1). This intervention program was a part of our community hypertension trial, and more detailed information is in the published study protocol [20].

In the baseline survey, we informed participants about the procedures of the intervention program, added them as friends in WeChat, and taught them how to finish the electronic questionnaire and correctly monitor BP. Based on 2010 Chinese guidelines for the management of hypertension [21], patients who had ≥3 risk factors (male aged >55 years or female aged >65 years, smoking, dyslipidemia, family history of premature cardiovascular disease, and Body Mass Index (BMI) ≥ 28 kg/m^2^) or clinical complications (cerebrovascular disease, heart disease, kidney disease, peripheral vascular disease, retinopathy, and diabetes) or SBP ≥ 180/DBP ≥ 110 mmHg were divided into the high cardiovascular risk group, and others were placed in the low–moderate-risk group. We built group chats for each risk group (approximately 30 individuals/group).

The intervention program consisted of four parts: health education, health promotion, group chat, and BP monitoring. First, we provided three months of health education via articles on WeChat Official accounts on common hypertension problems, medication treatment, complication prevention, and healthy lifestyle. After individuals read each article, they completed a short quiz (for example, “What are the diagnostic criteria for hypertension (mmHg)?” with four options (120/80, 135/85, 140/90, 160/100). In addition, at the end of each quiz, we would tell participants the correct answers with explanations to help them consolidate their knowledge. A three-month period of health promotion followed. Articles included how to take medicine, eat healthfully, exercise, quit smoking, limit alcohol, and regulate mood. A punch-in system was required to promote healthy behaviors. Throughout the whole intervention phase, we sent articles and quizzes in the group chat, and encouraged patients to ask questions, share personal experiences, report their BP, and discuss lifestyle topics. Each chat lasted at least one hour. They could also choose a private chat with researchers via WeChat messages at any time. For example, one patient who had a sudden rise in BP might ask for help in a private chat, and our community doctor would give some professional advice. We asked for self-reported BP at least once a week to track their BP and gave them some feedback.

The contents of articles sent to participants were all the same, but we separated one-week content into two parts to increase the frequency of the intervention for the high-risk group. The high-risk group had an intervention twice a week and the low-moderate-risk group had an intervention once a week.

The control group only received the usual community health care service, including health lectures and one chronic disease follow-up every three months.

### 2.4. Outcome Measures

Baseline and six-month follow-up surveys were completed in the clinic. BP was measured three times five minutes apart by an electronic automated sphygmomanometer (HEM-8713; Omron Corporation, Kyoto, Japan). Others were gathered by a self-completed questionnaire. Hypertension knowledge was a 35-item questionnaire designed by Chinese guidelines for hypertensive patients’ education [22]. It consisted of diagnostic criteria (1 item), salt intake (1 item), symptoms (9 items), complications (7 items), risk factors (8 items), medication (1 item), and self-management (8 items). The Cronbach’s α-coefficient was 0.688, indicating the acceptability of measuring the knowledge of hypertension. The Hypertension Self-Efficacy Scale was translated from an authoritative American scale and improved to be more suitable for Chinese respondents [23]. It is a 15-item scale that includes four dimensions: daily life (4 items), health behavior (6 items), medication adherence (3 items), and self-management (2 items). The questionnaire was proved to have good construct validity (explaining 79.77% of variance), content validity (r = 0.916), internal consistency (Cronbach’s α = 0.852), and test–retest reliability (r = 0.869). The Hypertension Patients Self-Management Behavior Rating Scale (HPSMBRS) [24] was designed to measure self-management. This 33-item scale consists of six subscales: medication adherence (4 items), condition monitoring (4 items), diet management (10 items), exercise management (3 items), work–rest management (5 items), and emotion management (7 items). The Cronbach’s α-coefficient was 0.914. The Social Support Rating Scale [25] was used to measure social support. This 10-item scale consists of subscales on subjective support (emotional experience), objective support, and use of social support.

The primary outcome was SBP change between baseline and follow-up. DBP, BP control, frequency of BP monitoring, hypertension knowledge, self-efficacy, self-management, and social support were analyzed as secondary outcomes.

### 2.5. Statistical Analysis

The sample size was calculated based on the difference in the mean SBP change between the intervention and control groups. We set the sample allocation ratio of the intervention and control groups to 1:1.5. According to our previous study [26], a 7.5 mmHg difference between two groups in Guangzhou hypertensive patients was reported, and we assumed an SD of 17 mmHg. Based on these assumptions, a sample size of 91 (intervention group) and 137 (control group) was expected to detect a 7.5 mmHg difference in SBP with a two-sided α of 0.05 and 90% power. Considering a slightly higher dropout rate of 50% for this mobile phone intervention, a total of 456 participants (182 participants in the intervention group and 274 participants in the control group) would be required.

Descriptive statistics, *t*-tests, and Chi-square tests were used to describe and compare the baseline demographic characteristics and outcomes between the intervention and control groups. The changes in primary and continuous secondary outcomes were compared using a linear mixed-effects model. We adjusted for demographics (sex, age, education, marriage, work status, and income), hypertension course, and comorbidities. We could not control for cluster sites, since the study included only two communities. The logistic mixed-effects model was performed for binary secondary outcomes (hypertension control and frequency of monitoring BP) to estimate the adjusted odds ratios between groups. The adjusted variables were the same as mentioned previously. All analyses were performed using R version 3.6.0 (R Foundation for Statistical Computing, Vienna, Austria). A two-sided *p*-value < 0.05 was considered statistically significant.

## 3. Results

**Figure 2:**
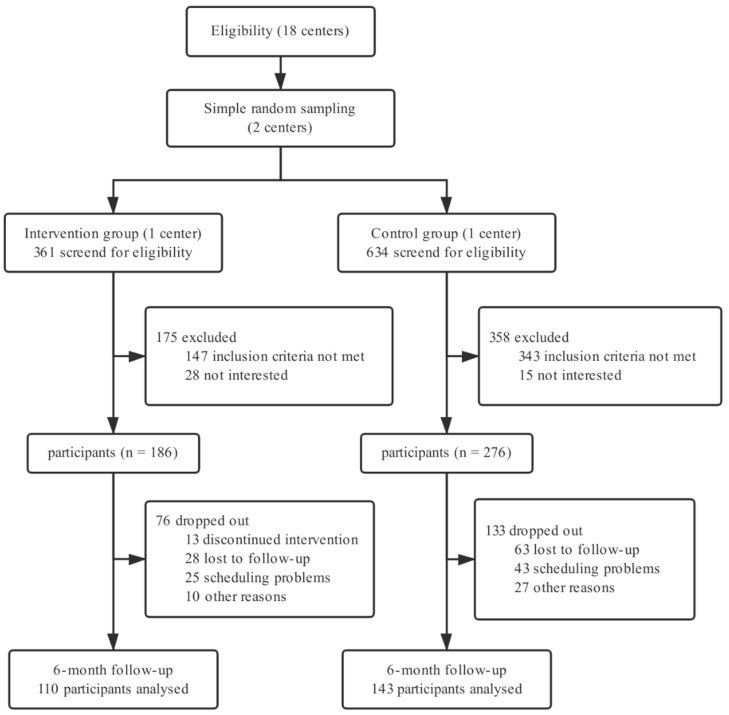
Study design and participants flow diagram.

Of the 995 individuals screened for eligibility, 533 (53.6%) were excluded: 490 (49.2%) did not meet the inclusion criteria and 43 (4.3%) were not interested (Figure 2). The main reasons for exclusion were not having hypertension (399 individuals), not using WeChat (53 individuals), and being overage (38 individuals). The remaining 462 (46.4%) patients were recruited and assigned to the intervention (*n* = 186) or control group (*n* = 276) according to the health care center allocation.

### 3.1. Baseline Characteristics

The mean age of the participants was 61.5 (6.4) years, 63.4% were female, 69.3% had a high school education or above, 86.1% were married, 14.9% were working, and 32.3% had low incomes (average household income <3000 RMB/month). The mean SBP was 135.4 (15.2) mmHg, and the DBP was 81.9 (10.5) mmHg. The baseline hypertension control rate was 64.9%.

There were no significant differences in baseline characteristics between the intervention and control groups (Table 1), other than intervention participants having higher education levels, incomes, disease knowledge scores, and social support scores.

### 3.2. Follow-Up of Participants

Overall, 253 participants (54.8%) completed the follow-up visits: 110 in the intervention group and 143 in the control group (Figure 2). There were no significant differences in the baseline characteristics between the missing and retained groups, except that poorly educated participants were more likely to drop out (*p* = 0.044).

### 3.3. Primary and Secondary Outcomes

Table 2 shows the mean pre/post changes in SBP and DBP between the intervention and control groups. SBP declined obviously in the intervention group after the six-month intervention program. Relatively, there were no significant differences in SBP between baseline and follow-up in the control group. In general, BP changed significantly in the intervention group compared to the control group: differences in adjusted mean SBP and DBP changes were −6.9 (95% CI −11.2 to −2.6; *p* = 0.002) and −3.1 (95% CI −5.7 to −0.6; *p* = 0.016) mmHg, respectively. In addition, compared to the control group, hypertension control changed significantly (adjusted odds ratio: 5.0 (2.3, 11.3); *p* < 0.001) in the intervention group, with the rate increasing from 60.9% to 83.6% (Table 3).

A total of 78.1% of the intervention participants monitored BP at least once a week after our six-month intervention program (Table 3). The BP monitoring rate changed significantly compared to the control (adjusted odds ratio: 4.2 (1.8, 11.3); *p* < 0,001).

Changes in hypertension knowledge, self-efficacy, and social support scores did not differ between the two groups. However, the intervention participants’ scores of some subscales in hypertension knowledge and self-efficacy scale did rise slightly in comparison with the scores of the control participants. The adjusted mean differences in risk factors and methods of self-management from hypertension knowledge were 0.9 (95% CI 0.1 to 1.7; *p* = 0.027) and 0.8 (95% CI 0.2 to 1.5; *p* = 0.009), respectively. In addition, daily life in self-efficacy had a 0.8 (95% CI 0.1 to 1.5; *p* = 0.025) adjusted mean difference between groups. Additionally, the adjusted mean differences in self-management and its three dimensions (condition monitoring, diet management, and emotion management) were 8.7 (95% CI 4.7 to 12.7; *p* < 0.001), 1.6 (95% CI 0.7 to 2.5; *p* < 0.001), 4.2 (95% CI 2.7 to 5.6; *p* < 0.001), and 1.9 (95% CI 0.3 to 3.4; *p* = 0.019), respectively. Table 4 shows all the items for each scale. There was no evidence of improved scores in hypertension knowledge (diagnostic criteria, symptoms, and complications) or on the self-efficacy scale (health behavior, medication adherence, and self-management), the self-management scale (medication adherence, exercise management, and work-rest management), and the social support scale (subjective support, objective support, and use of social support).

## 4. Discussion

In this study, there was a significantly greater decline in BP between the intervention and control groups. The intervention group achieved a higher hypertension control rate and BP monitoring rate from baseline to follow-up. Individuals who attended the intervention program improved their hypertension self-management and parts of their disease knowledge and self-efficacy. Overall, the WeChat-based self-management intervention succeeded in the management of community middle-aged and elderly hypertensive patients in Guangzhou, China.

The clinical outcomes of this study were similar to those of other works [5,9], with significant improvements. In addition, previous studies [27,28,29] showed that lowering BP could reduce the risk of stroke and ischemic heart disease. A meta-analysis found that a 10-mmHg reduction in SBP or 5-mmHg reduction in DBP could reduce the incidence of stroke by 40% and ischemic heart disease by 22% [29]. The absolute reduction in BP (6.9/3.1 mmHg) was expected to cause an approximate decline of 25% in stroke risk and a 10% decline in ischemic heart disease risk in our trial. Although more than half of the participants’ BPs were under standard control at baseline, they were still in need of intensive BP control if the condition allowed. A recent intensive intervention trial [30] demonstrated that lower BP targets benefitted the whole population with hypertension.

How did our self-management intervention work among middle-aged and elderly hypertensive patients in the community? One possible mechanism for the effect might be that asking for self-reported BP and feedback increased the frequency of monitoring BP in the intervention group. We found that 78.1% of the individuals participating in the intervention measured their BP at least once a week at follow-up, which was significantly more frequently than the control group. Monitoring BP could increase patients’ awareness of their disease condition and lead them to make some positive behavioral changes. A meta-analysis [8] confirmed that self-monitoring combined with other interventions (e.g., education, medication titration, or lifestyle advising) was associated with lower BP.

A second possible mechanism might be that health education improved the hypertension knowledge of intervention participants; therefore, they understood the disease better, had more confidence in self-management, and made some behavior changes. Despite no significant difference in the total score change in hypertension knowledge, the scores for sub-problems (risk factors and methods of self-management) did improve significantly. This might be due to the intervention group having higher scores at baseline than did the control group. Among the items of risk factors, patients had relatively less knowledge of smoking, alcohol, high-fat diet, and stress at baseline. In addition, self-efficacy did not increase, which might have been due to a ceiling effect. One dimension of daily life (including four items: reducing worry or anxiety in life, controlling weight, avoiding working too much, and exercising more) significantly improved in the intervention group, indicating that patients had more confidence in emotion management, weight control, work–rest management, and exercise, all of which might gradually change their daily lifestyle.

A third possible mechanism might be that health promotion improved patients’ self-management to decrease their BP. Chronic disease self-management encompasses living with a disease using strategies such as controlling symptoms, addressing physical conditions, focusing on inherent physical and mental states, and changing lifestyles. Patients with higher self-management skills will be more likely to achieve disease control. Our work confirmed that the intervention group, with increasing self-management, did develop better BP control. Due to the ceiling effect, we only found a difference in three dimensions (condition monitoring, diet management, and emotion management).

A fourth possible mechanism might be that the group chat made a positive group environment for patients sharing personal self-management experiences, and the private chat provided personalized advice for patients, which would improve their confidence in disease control and which would be conducive to the decline in BP. Participants in the intervention group did indeed gain social support from other patients in the same chat group and from health workers during the six-month intervention. An eHealth self-management program in Korean [31] showed great improvement in social support, while the score of social support with no difference between groups might be due to the scale focusing more on support from relatives and friends. In general, based on the self-efficacy theory, our intervention could reduce BP through these four possible mechanisms. A further path analysis may be needed for better intervention design.

Primary health services from community health care centers are the most important part of chronic disease management in China. However, a large number of residents with chronic diseases increase the management burden for these community centers. The emergence of mHealth will make it more convenient for community health care centers to provide health services and disease management. Our study provided a self-management intervention for community hypertension patients via WeChat in Guangzhou, China, which lowered patients’ BP and improved their self-management. The intervention could serve as a good example for community health care centers in managing hypertensive patients registered in centers in Guangzhou and other similar cities, such as Beijing and Shanghai, which are first-tier cities in China with developed economy, primary health care services, and high standard of living. In the future, with economic, technological, and medical developments in China, the intervention based on WeChat may be expanded to the whole country. In addition, a study [32] with cost-effectiveness analysis demonstrated that self-management was the optimal form of hypertension management in the community centers.

Moreover, in recent years, family-doctor-contracted services have been put into practice in many community health care centers in China, whereas each community center is still struggling to manage patients with chronic disease among family doctors. Research in Shanghai, China [33] reported that group management achieved effective results for self-management in noncommunicable diseases. It is possible that our group-based self-management intervention via WeChat could be a more feasible and accessible method for family doctors. In addition to the rapid development of artificial intelligence [34], a more practical question–answer robot based on speech recognition and natural language processing technologies could improve the self-management of patients and reduce human costs.

This study had several limitations. First, there were only two community health care centers as clusters in the randomization for the intervention and control groups. Although most variables at baseline had no significant difference between the two groups, and although we adjusted all measured characteristics when performing outcome analysis, there were still many unmeasured covariates that could not be avoided. Second, both participants and researchers alike were unmasked to the group assignment in this study. We used an automatic sphygmomanometer to measure BP and self-administered questionnaires, yet we might overestimate the results of the scales. Third, 45.2% of participants failed to attend the follow-up survey, although there were no differences between the missing and retained groups except for in education. Finally, we only performed a six-month follow-up visit; thus, we could not ensure that the BP reduction would continue to the future. In summary, this study confirmed the WeChat-based self-management intervention’s practicability and availability in the short term. A better-designed trial with more clusters and long-term follow-up is needed to further examine the intervention.

## 5. Conclusions

In summary, this study found that a WeChat-based self-management intervention would be helpful for middle-aged and elderly hypertensive patients in the community aiming to lower their BP and achieve better self-management in Guangzhou, China. It may be a better form of technology for family doctors to use to manage hypertension in Chinese community health centers in the future. Future work should develop more applications of mHealth for the management of various diseases.

## Figures and Tables

**Table 1:** Baseline characteristics of participants. BP: blood pressure; DBP: diastolic BP; SBP: systolic BP.

Variables	Intervention (*n* = 186)	Control (*n* = 276)	*p*
Male	75 (40.3%)	94 (34.1%)	0.170
Age (years)	61.7 (6.3)	61.3 (6.4)	0.531
Education			<0.001
Secondary school or below	47 (25.3%)	95 (34.4%)	
High school	80 (43.0%)	136 (49.3%)	
College or above	59 (31.7%)	45 (16.3%)	
Married	163 (87.6%)	235 (85.1%)	0.447
Employed	31 (16.7%)	38 (13.8%)	0.391
Low income (<¥3000/month)	42 (22.6%)	107 (38.8%)	<0.001
Disease course (years)	9.2 (7.2)	9.0 (7.9)	0.825
Clinical complications			
Heart disease	28 (15.1%)	37 (13.4%)	0.617
Cerebrovascular disease	6 (3.2%)	7 (2.5%)	0.660
Kidney disease	8 (4.3%)	7 (2.5%)	0.294
Diabetes	34 (18.3%)	59 (21.4%)	0.416
Family history of hypertension	150 (80.6%)	206 (74.6%)	0.132
Monitoring BP	107 (57.5%)	159 (57.6%)	0.986
SBP (mmHg)	135.8 (15.9)	135.2 (14.8)	0.679
DBP (mmHg)	83.0 (10.1)	81.1 (10.8)	0.056
Hypertension control	113 (60.8%)	187 (67.8%)	0.148
Disease knowledge	22.9 (6.6)	19.7 (6.6)	<0.001
Self-efficacy	66.1 (5.6)	66.5 (6.4)	0.551
Self-management	138.1 (14.9)	142.3 (14.9)	0.003
Social support	39.4 (6.1)	37.0 (6.7)	<0.001

Data are mean (SD) or n (%).

**Table 2:** Mean changes (95% CI) in SBP and DBP.

	Mean Difference from Baseline	Difference between Groups	*p*
SBP (mmHg); unadjusted			
Intervention	−5.5 (−8.3, −2.7)	−7.1(−11.4, −2.8)	0.001
Control	1.6 (−1.2, 4.4)		
SBP (mmHg); adjusted *			
Intervention	−5.3 (−8.2, −2.4)	−6.9 (−11.2, −2.6)	0.002
Control	1.6 (−1.2, 4.5)		
DBP (mmHg); unadjusted			
Intervention	−1.3 (−3.0, 0.4)	−3.4 (−5.9, −0.8)	0.011
Control	2.1 (0.4, 3.8)		
DBP (mmHg); adjusted *			
Intervention	−1.1 (−2.7, 0.6)	−3.1 (−5.7, −0.6)	0.016
Control	2.0 (0.6, 3.7)		

* Adjusted for sex, age, education, marriage, work status, income, disease course, and clinical complications.

**Table 3:** Odds ratios (95% CI) in hypertension control and monitoring BP.

	Baseline	Follow-up	Adjusted Odds Ratio *	*p*
Hypertension control (SBP < 140, DBP < 90 mmHg)				
Intervention	67 (60.9%)	92 (83.6%)	5.0 (2.3, 11.3)	<0.001
Control	99 (69.2%)	91 (63.6%)		
Monitoring BP (≥1/week)				
Intervention	63 (57.3%)	86 (78.1%)	4.2 (1.8, 10.1)	0.001
Control	84 (58.7%)	81 (56.6%)		

* Adjusted for sex, age, education, marriage, work status, income, disease course, and clinical complications.

**Table 4:** Mean changes (95%CI) in hypertension knowledge, self-efficacy, self-management, and social support.

Outcomes (Aggregate Score)	Intervention	Control	Adjusted Mean Difference *	*p*
**Hypertension knowledge (35)**	2.3 (0.8, 3.8)	0.8 (−0.4, 2.0)	1.5 (−0.3, 3.3)	0.110
Diagnostic criteria (1)	0.2 (0.1, 0.3)	0.1 (0.0, 0.2)	0.1 (−0.1, 0.2)	0.218
Salt intake (1)	0.1 (0.0,0.2)	0.1 (0.0, 0.2)	0.0 (−0.1, 0.1)	0.585
Symptoms (9)	0.6 (0.3, 1.0)	0.7 (0.4, 1.1)	−0.1 (−0.7, 0.5)	0.747
Complications (7)	0.6 (0.0, 1.1)	−0.2 (−0.8, 0.3)	0.8 (0.0, 1.7)	0.055
Risk factors (8)	0.6 (0.0, 1.2)	−0.3 (−0.8, 0.3)	0.9 (0.1, 1.7)	0.027
Methods of self-management (8)	0.5 (0.0, 1.0)	−0.3 (−0.7, 0.1)	0.8 (0.2, 1.5)	0.009
**Self-efficacy (75)**	0.8 (−0.4, 2.0)	−0.6 (−1.7, 0.4)	1.4 (−0.2, 3.0)	0.086
Daily life (20)	0.6 (0.1, 1.1)	−0.2 (−0.6, 0.3)	0.8 (0.1, 1.5)	0.025
Health behavior (30)	0.0 (−0.4, 0.5)	−0.5 (−0.9, 0.0)	0.5 (−0.2, 1.3)	0.153
Medication adherence (15)	0.0 (−0.4, 0.4)	0.2 (−0.2, 0.6)	−0.2 (−0.8, 0.4)	0.615
Self-management (10)	0.2 (−0.2, 0.5)	−0.1 (−0.5, 0.2)	0.3 (−0.2, 0.8)	0.242
**Self-management (165)**	7.3 (4.3, 10.3)	−1.4 (−4.0, 1.2)	8.7 (4.7, 12.7)	<0.001
Medication adherence (20)	0.2 (−0.3, 0.7)	−0.2 (−0.7, 0.3)	0.4 (−0.4, 1.2)	0.355
Condition monitoring (20)	1.6 (1.0, 2.2)	0.0 (−0.6, 0.6)	1.6 (0.7, 2.5)	<0.001
Diet management (50)	1.7 (0.6, 2.8)	−2.5 (−3.5, −1.6)	4.2 (2.7, 5.6)	<0.001
Exercise management (15)	1.3 (0.5, 2.0)	0.8 (0.0, 1.5)	0.5 (−0.7, 1.7)	0.389
Work–rest management (25)	1.9 (0.7, 3.0)	0.4 (−0.8, 1.5)	1.5 (−0.2, 3.2)	0.091
Social support (64)	0.4 (−0.1, 0.9)	0.7 (0.3, 1.2)	−0.3 (−1.1, 0.3)	0.309
Subjective support (20)	0.0 (−0.5, 0.5)	0.2(−0.3, 0.7)	−0.2 (−0.8, 0.4)	0.521
Objective support (32)	−0.1 (−0.6, 0.4)	0.1 (−0.4, −0.6)	−0.2 (−1.0, 0.7)	0.647
Use of social support (12)	0.5 (0.0, 1.0)	0.6 (0.1, 1.1)	−0.1 (−0.9, 0.7)	0.785

* Adjusted for sex, age, education, marriage, work status, income, disease course, and clinical complications.

## References

[1] Lim S.S., Vos T., Flaxman A.D., Danaei G., Shibuya K., Adair-Rohani H., Amann M., Anderson H.R., Andrews K.G., Aryee M. (2012). A comparative risk assessment of burden of disease and injury attributable to 67 risk factors and risk factor clusters in 21 regions, 1990–2010: A systematic analysis for the Global Burden of Disease Study 2010. Lancet.

[2] Zhou M., Wang H., Zeng X., Yin P., Zhu J., Chen W., Li X., Wang L., Wang L., Liu Y. (2019). Mortality, morbidity, and risk factors in China and its provinces, 1990–2017: A systematic analysis for the Global Burden of Disease Study 2017. Lancet.

[3] Wang Z., Chen Z., Zhang L., Wang X., Hao G., Zhang Z., Shao L., Tian Y., Dong Y., Zheng C. (2018). Status of Hypertension in China: Results from the China Hypertension Survey, 2012–2015. Circulation.

[4] Liu L., Wu S., Wang J., Wang W., Bao Y., Cai J., Chen L., Chen W., Chu S., Feng Y. (2019). 2018 Chinese guidelines for the management of hypertension. Chin. J. Cardiol..

[5] McManus R., Mant J., Bray E.P., Holder R., Jones M.I., Greenfield S., Kaambwa B., Banting M., Bryan S., Little P. (2010). Telemonitoring and self-management in the control of hypertension (TASMINH2): A randomised controlled trial. Lancet.

[6] Uhlig K., Patel K., Ip S., Kitsios G.D., Balk E.M. (2013). Self-measured blood pressure monitoring in the management of hypertension: A systematic review and meta-analysis. Ann. Intern. Med..

[7] Qureshi N.N., Hatcher J., Chaturvedi N., Jafar T.H., Hypertension Research G. (2007). Effect of general practitioner education on adherence to antihypertensive drugs: Cluster randomised controlled trial. BMJ.

[8] Tucker K.L., Sheppard J.P., Stevens R., Bosworth H.B., Bove A., Bray E.P., Earle K., George J., Godwin M., Green B.B. (2017). Self-monitoring of blood pressure in hypertension: A systematic review and individual patient data meta-analysis. PLoS Med..

[9] Margolis K.L., Asche S.E., Bergdall A.R., Dehmer S.P., Groen S.E., Kadrmas H.M., Kerby T.J., Klotzle K.J., Maciosek M.V., Michels R.D. (2013). Effect of home blood pressure telemonitoring and pharmacist management on blood pressure control: A cluster randomized clinical trial. JAMA.

[10] McManus R.J., Mant J., Franssen M., Nickless A., Schwartz C., Hodgkinson J., Bradburn P., Farmer A., Grant S., Greenfield S.M. (2018). Efficacy of self-monitored blood pressure, with or without telemonitoring, for titration of antihypertensive medication (TASMINH4): An unmasked randomised controlled trial. Lancet.

[11] Watson A.J., Singh K., Myint U.K., Grant R.W., Jethwani K., Murachver E., Harris K., Lee T.H., Kvedar J.C. (2012). Evaluating a web-based self-management program for employees with hypertension and prehypertension: A randomized clinical trial. Am. Heart J..

[12] Hui C.Y., Creamer E., Pinnock H., McKinstry B. (2019). Apps to Support Self-Management for People with Hypertension: Content Analysis. JMIR Mhealth Uhealth.

[13] Alessa T., Hawley M.S., Hock E.S., de Witte L. (2019). Smartphone Apps to Support Self-Management of Hypertension: Review and Content Analysis. JMIR Mhealth Uhealth.

[14] Number of Monthly Active WeChat Users from 2nd Quarter 2012 to 2nd Quarter 2019 (in Millions). https://www.statista.com/statistics/255778/number-of-active-wechat-messenger-accounts/.

[15] Xia J., Hu S., Xu J., Hao H., Yin C., Xu D. (2018). The correlation between glucose fluctuation from self-monitored blood glucose and the major adverse cardiac events in diabetic patients with acute coronary syndrome during a 6-month follow-up by WeChat application. Clin. Chem. Lab. Med..

[16] He C., Wu S., Zhao Y., Li Z., Zhang Y., Le J., Wang L., Wan S., Li C., Li Y. (2017). Social Media-Promoted Weight Loss Among an Occupational Population: Cohort Study Using a WeChat Mobile Phone App-Based Campaign. J. Med. Internet Res..

[17] Hou L., Jin X., Ma J., Qian J., Huo Y., Ge J. (2019). Perception and self-management of hypertension in Chinese cardiologists (CCHS): A multicenter, large-scale cross-sectional study. BMJ Open.

[18] Wang J.G. (2018). Unique approaches to hypertension control in China. Ann. Transl. Med..

[19] Bandura A. (1977). Self-efficacy: Toward a unifying theory of behavioral change. Psychol. Rev..

[20] Li T., Ding W.W., Li X.W., Lin A.H. (2019). Mobile health technology (WeChat) for the hierarchical management of community hypertension: Protocol for a cluster randomized controlled trial. Patient Prefer. Adher..

[21] Writing Group of 2010 Chinese Guidelines for the Management of Hypertension (2011). 2018 Chinese guidelines for the management of hypertension. Chin. J. Cardiol..

[22] Wu Z., Huo Y., Wang W., Zhao L., Zhu D. (2014). Chinese Guidelines for Patient Education of Hypertension. Chin. J. Front. Med. Sci. (Electron. Vers.).

[23] Chen J. (2011). Analysis and Intervention of Knowledge, Belief, Behavior in Elderly Patients with Primary Hypertension in Haizhu District of Guangzhou.

[24] Zhao Q., Liu X. (2012). Reliability and validity of the hypertension patients self-management behavior rating scale. Chin. Nurs. Manag..

[25] Lan G., Yuan Z., Cook A., Xu Q., Jiang H., Zheng H., Wang L., Yuan L., Xie X., Lu Y. (2015). The relationships among social support and quality of life in persons living with HIV/AIDS in Jiangxi and Zhejiang provinces, China. AIDS Care.

[26] Lin A., Zhang G., Liu Z., Gu J., Chen W., Luo F. (2014). Community-based lifestyle intervention for reducing blood pressure and glucose among middle-aged and older adults in China: A pilot study. Int. J. Environ. Res. Public Health.

[27] Collins R., Peto R., MacMahon S., Hebert P., Fiebach N.H., Eberlein K.A., Godwin J., Qizilbash N., Taylor J.O., Hennekens C.H. (1990). Blood pressure, stroke, and coronary heart disease. Part 2, Short-term reductions in blood pressure: Overview of randomised drug trials in their epidemiological context. Lancet.

[28] Staessen J.A., Gasowski J., Wang J.G., Thijs L., Den Hond E., Boissel J.P., Coope J., Ekbom T., Gueyffier F., Liu L. (2000). Risks of untreated and treated isolated systolic hypertension in the elderly: Meta-analysis of outcome trials. Lancet.

[29] Law M.R., Morris J.K., Wald N.J. (2009). Use of blood pressure lowering drugs in the prevention of cardiovascular disease: Meta-analysis of 147 randomised trials in the context of expectations from prospective epidemiological studies. BMJ.

[30] Group S.R., Wright J.T., Williamson J.D., Whelton P.K., Snyder J.K., Sink K.M., Rocco M.V., Reboussin D.M., Rahman M., Oparil S. (2015). A Randomized Trial of Intensive versus Standard Blood-Pressure Control. N. Engl. J. Med..

[31] Jung H., Lee J.E. (2017). The impact of community-based eHealth self-management intervention among elderly living alone with hypertension. J. Telemed. Telecare.

[32] Zhang X., Liao H., Shi D., Li X., Chen X., He S. (2019). Cost-effectiveness analysis of different hypertension management strategies in a community setting. Intern. Emerg. Med..

[33] Huang J., Zhang T., Wang L., Guo D., Liu S., Lu W., Liang H., Zhang Y., Liu C. (2019). The effect of family doctor-contracted services on noncommunicable disease self-management in Shanghai, China. Int. J. Health Plann. Manag..

[34] Esteva A., Robicquet A., Ramsundar B., Kuleshov V., DePristo M., Chou K., Cui C., Corrado G., Thrun S., Dean J. (2019). A guide to deep learning in healthcare. Nat. Med..

